# Transcriptome Analysis Provides Insights Into the Adaptive Responses to Hypoxia of a Schizothoracine Fish (*Gymnocypris eckloni*)

**DOI:** 10.3389/fphys.2018.01326

**Published:** 2018-09-21

**Authors:** Delin Qi, Yan Chao, Rongrong Wu, Mingzhe Xia, Qichang Chen, Zhiqin Zheng

**Affiliations:** ^1^State Key Laboratory of Plateau Ecology and Agriculture, Qinghai University, Xining, China; ^2^Animal Science Department, Agriculture and Animal Husbandry College, Qinghai University, Xining, China

**Keywords:** *Gymnocypris eckloni*, RNA-Seq, hypoxia, transcriptome, gene, pathway

## Abstract

The schizothoracine fish endemic to the Qinghai-Tibetan Plateau are comparatively well adapted to aquatic environments with low oxygen partial pressures. However, few studies have used transcriptomic profiling to investigate the adaptive responses of schizothoracine fish tissues to hypoxic stress. This study compared the transcriptomes of *Gymnocypris eckloni* subjected to 72 h of hypoxia (Dissolved oxygen, DO = 3.0 ± 0.1 mg/L) to those of *G. eckloni* under normoxia (DO = 8.4 ± 0.1 mg/L). To identify the potential genes and pathways activated in response to hypoxic stress, we collected muscle, liver, brain, heart, and blood samples from normoxic and hypoxic fish for RNA-Seq analysis. We annotated 337,481 gene fragments. Of these, 462 were differentially expressed in the hypoxic fish as compared to the normoxic fish. Under hypoxia, the transcriptomic profiles of the tissues differed, with muscle the most strongly affected by hypoxia. Our data indicated that *G. eckloni* underwent adaptive changes in gene expression in response to hypoxia. Several strategies used by *G. eckloni* to cope with hypoxia were similar to those used by other fish, including a switch from aerobic oxidation to anaerobic glycolysis and the suppression of major energy-requiring processes. However, *G. eckloni* used an additional distinct strategy to survive hypoxic environments: a strengthening of the antioxidant system and minimization of ischemic injury. Here, we identified several pathways and related genes involved in the hypoxic response of the schizothoracine fish. This study provides insights into the mechanisms used by schizothoracine fish to adapt to hypoxic environments.

## Introduction

Hypoxia is an important environmental factor that affects the metabolism and survival of most aerobic animals. Aqueous environments are characterized by low oxygen capacitance and poor oxygen diffusibility; these challenges may be increased by human activities (Diaz, 2001). Fish living in environments with low and/or variable oxygen supplies have evolved complex suites of biochemical, physiological, behavioral, and molecular adaptations that enable survival under such conditions (Gracey et al., 2001; Nikinmaa, 2002; Nikinmaa and Rees, 2005; Roesner et al., 2006, 2008; Wang et al., 2017; Liu et al., 2018). Changes in transcription profiles in response to hypoxia have been investigated in several fish taxa, including the hypoxia-tolerant goby fish, *Gillichthys mirabilis* (Gracey et al., 2001); zebrafish (*Danio rerio*) (Ton et al., 2003; van der Meer et al., 2005); viviparous (*Xiphophorus*) and oviparous (*Oryzias*) fish (Boswell et al., 2009); *Carassius auratus* (Zhong et al., 2009; Liao et al., 2013); large yellow croaker (*Larimichthys crocea*) (Wang et al., 2017); and Nile tilapia (*Oreochromis niloticus*) (Liu et al., 2018; Xia et al., 2018). Fish exhibit a variety of adaptive responses to hypoxia, including the discontinuation of processes requiring substantial energy output, such as protein synthesis (Gracey et al., 2001; Ton et al., 2003), locomotion (Gracey et al., 2001), and cell growth/proliferation (Gracey et al., 2001). Hypoxia also stimulates the antioxidant system (Zhong et al., 2009; Wang et al., 2017), and induces the expression of immune-related genes (Wang et al., 2017) and genes associated with lysosomal lipid trafficking and degradation (van der Meer et al., 2005). However, the molecular mechanisms underlying the response of schizothoracine fish to hypoxic stress remain unclear.

Schizothoracine fish (Teleostei: Cyprinidae) are the largest and most diverse taxon within the Qinghai-Tibetan Plateau (QTP) ichthyofauna (Wu and Wu, 1992; Chen and Cao, 2000). The schizothoracine fish are divided into three groups based on morphological characters: primitive, specialized, and highly specialized (Cao et al., 1981). Previous studies have demonstrated that schizothoracine fish restricted to high-altitude or high-latitude environments are well adapted to cold, hypoxic conditions (Qi et al., 2012; Li et al., 2013; Guan et al., 2014; Xia et al., 2016). Therefore, the schizothoracine fish are an excellent model for studies of the fundamental mechanisms of adaptions to hypoxia and low temperatures. *Gymnocypris eckloni*, is a representative species of the highly specialized schizothoracine fish, and this species is widely distributed in isolated lakes and the upper reaches of the Yellow River (Wu and Wu, 1992; Chen and Cao, 2000). This species is very well adapted to the dissolved oxygen fluctuations of its aqueous environment (Qi et al., 2012; Xia et al., 2016). During artificial breeding experiments, *G. eckloni* displayed a substantial tolerance for hypoxia, surviving acute hypoxia (∼0.3 mg O_2_/L) for 12 h and chronic hypoxia (∼3.0 mg O_2_/L) for much longer (Xia et al., 2016). Therefore, we hypothesized that *G. eckloni* would exhibit transcriptional responses to hypoxia that would differ from less hypoxia-tolerant fish species.

Understanding changes in the gene expression in fish exposed to hypoxia might reveal new mechanisms of hypoxic acclimation, and might shed light on the evolution of this adaptive response in vertebrates. In the present study, we sequenced the transcriptomes of five tissues (the liver, muscle, brain, heart, and blood) from *G. eckloni* under normoxic or hypoxic conditions. We used this transcriptomic data to examine the gene expression profiles of *G. eckloni* exposed to hypoxia. Our results increase our understanding of the adaptive mechanisms used by the schizothoracine fish in response to hypoxia.

## Materials and Methods

### Experimental Fish and Hypoxia Treatment

Healthy *G. eckloni* (average body weight: ∼170 g) were obtained from the Native Fish Artificial Proliferation and Release Station, Xunhua, Qinghai Province, China. Fish were transported to our laboratory in aerated buckets. We used six adult individuals for our experiments. All of the fish were allowed to acclimate for 3 days before experimentation, in 10–13°C freshwater almost identical in composition to the river water at the capture site. All of the research involving animals followed the guidelines of, and was conducted under the approval of, the Animal Care and Use Committee, Qinghai University, China.

Three fish were randomly assigned to the control group (normoxia); the remaining three fish were assigned to the hypoxia group. The two groups were kept in separate, identical 20 L translucent plastic tanks. The freshwater in the tanks was bubbled with nitrogen and with the ambient air in our laboratory. The dissolved oxygen (DO) level was continuously monitored using an AZ8402 dissolved oxygen meter (AZ Instrument Corp., Taiwan) (Xia et al., 2016). In the normoxia (control) tank, DO was 8.4 ± 0.1 mg/L. In the hypoxia tank, DO was reduced from 8.4 ± 0.1 to 3.0 ± 0.1 mg/L over 1 h, and then maintained at 3.0 ± 0.1 mg/L for 72 h.

After the experiment, fish were stunned with a blow to the head and blood was quickly collected from a caudal puncture in an EDTA-treated tube, then transferred to a microfuge tube. The liver, brain, and heart were removed from each fish, and a sample was taken of the muscle tissue. Blood and tissue samples were stored in liquid nitrogen until RNA isolation.

### RNA Extraction, Illumina Library Preparation, and Sequencing

An Ambion MagMAX-96 total RNA isolation kit (Life Sciences, United States) was used to isolate total RNA from each sample, following the manufacturer’s instructions. RNA degradation and contamination were monitored on 1% agarose gels. RNA purity was analyzed with a NanoPhotometer Spectrophotometer (Implen, United States). The RNA concentration was quantified with a Qubit RNA Assay Kit in a Qubit 2.0 Fluorometer (Life Technologies, United States). RNA integrity was analyzed using a RNA Nano 6000 Assay Kit and an Agilent Bioanalyzer 2100 (Agilent Technologies, United States). Thirty separate Illumina sequencing libraries were prepared using 3.0 μg RNA per sample.

Sequencing libraries were created with the NEBNext Ultra RNA Library Prep Kit for Illumina (NEB, United States), following the manufacturer’s instructions. Index codes were added to assign sequences for each sample. In brief, mRNA was purified from the total RNA with poly-T oligo-attached magnetic beads. Fragmentation was done with divalent cations under an elevated temperature in NEBNext First Strand Synthesis Reaction Buffer (NEB, United States). First strand cDNA was synthesized with M-MuLV Reverse Transcriptase (RNase H^-^) and random hexamer primers. Second strand cDNA synthesis was subsequently carried out with RNase H and DNA Polymerase I. Exonuclease/polymerase activity converted the remaining overhangs into blunt ends. After adenylation of the 3′ ends of the DNA fragments, NEBNext Adaptors with hairpin loop structures were ligated to prepare for hybridization. Library fragments were purified with the AMPure XP system (Beckman Coulter, United States) to preferentially select 150–200 bp cDNA fragments. The adaptor-ligated, size-selected cDNA was then incubated with 3.0 μl USER Enzyme (NEB, United States) for 15 min at 37°C followed by 5 min at 95°C before PCR. PCRs were performed using universal PCR primers, Phusion High-Fidelity DNA polymerase, and the Index (X) Primer. Finally, PCR products were purified (with the AMPure XP system). Library quality was assessed with an Agilent Bioanalyzer 2100.

Clustering of the index-coded samples was carried out on a cBot Cluster Generation System using a TruSeq PE Cluster Kit v3-cBot-HS (Illumina), following the manufacturer’s instructions. Library preparations were sequenced on an Illumina HiSeq 2500 platform after cluster generation, which generated 125 bp paired-end reads. All data sets have been submitted to the National Center for Biotechnology Information (NCBI) Sequence Read Archive (SRA) database (accession number SRP150490).

### Quality Control and *de novo* Assembly

Raw data (raw reads) in FASTQ format were sorted by individual fish and processed using self-written Perl scripts. Our scripts generated cleaned data (cleaned reads) by removing reads containing adapter sequences, reads containing poly-N, and low quality reads. We also calculated Q20, Q30, GC-content, and sequence duplication level for the cleaned data. All of the downstream analyses were based on high-quality cleaned data.

*De novo* transcriptome assembly was performed using the short read assembly program Trinity, with min_kmer_cov set to 2 by default and all of the other parameters set default (Grabherr et al., 2011). Briefly, reads of a certain length with overlapping areas were joined initially in order to form longer fragments (contigs) without gaps. The paired-end reads were then mapped back onto the contigs, which were subsequently connected. These steps were repeated until the sequences could no longer be extended (Stewart et al., 2015). The final sequences were considered to be unigenes, which still were, strictly speaking, gene fragments.

### Transcriptome Annotation

We annotated the gene fragments against the NCBI non-redundant (Nr) protein database, the Swiss-Prot database, the Kyoto Encyclopedia of Genes and Genomes (KEGG) database (Kanehisa et al., 2004), the Clusters of Orthologous Groups of proteins (COG) database, the Pfam database, and the nucleotide (Nt) database using BLASTX, with an *E*-value 1.0 × 10^-5^. All of the annotated gene fragments were translated into amino acid sequences using ESTScan (Iseli et al., 1999), and then screened against the Gene Ontology (GO) database using Blast2GO (Conesa et al., 2005). The clusters of orthologous groups (COG) database was used to identify the putative functions of each gene fragment product based on known orthologous gene products (Tatusov et al., 2003). KEGG pathways were identified using the KEGG Automatic Annotation Sever (KAAS^1^) with the bi-directional best-hit method and an *E*-value of 1.0 × 10^-10^.

### Differential Expression Analysis and Functional Enrichment

To test for differentially expressed genes (DEGs), individual sequence reads from each sample were mapped back to the assembled transcriptome using the alignment program Bowtie (Langmead et al., 2009). Differential expression analysis was performed using the DESeq R package (Anders and Huber, 2010). This package includes statistical routines that determine differential expressions in digital gene expression data based on a model of a negative binomial distribution. Mean expression levels were generated using three tissue samples per treatmen. The resulting *P*-values were adjusted using the Benjamini-Hochberg approach for controlling the false discovery rate. Genes identified by DEGSeq with an adjusted *P*-value < 0.05 were considered DEGs. GO and KEGG pathway analyses were then performed on the DEGs.

### Quantitative Real-Time PCR Validation (qRT-PCR)

RNA samples from three biological replicates were used for qRT-PCR validation of the transcriptome data. RNA used for transcriptomic sequencing was converted into cDNA using the RNAprep Pure Tissue Kit (TIANGEN Biotech Co., Ltd., China), and treated with RNase-free DNase I (Takara, Japan). Gene-specific primers were designed using Primer Premier 5.0 (**Supplementary Table 1**). qRT-PCR was performed in an iQ5 Multicolor Real-Time PCR Detection System (Bio-Rad Laboratories, Inc., United States) using SuperReal PreMix Plus (SYBR Green) (TIANGEN Biotech Co., Ltd.). All of the samples were analyzed in triplicate (as technical replicates) and fold changes in gene expression were calculated using the 2^-ΔΔCt^ method (Livak and Schmittgen, 2001). GAPDH served as the internal control.

## Results

### Raw Sequencing Data and *de novo* Transcriptome Assembly

Here, 30 separate Illumina sequencing libraries produced close to 1,837.7 million paired-end reads (2 × 125 bp). After trimming and quality filtering, 4.5% of all of the reads were discarded, leaving over 1,753.7 million reads (219.12 Gb) for downstream analysis. The number of qualified Illumina reads per sample ranged from 50.9 million (6.36 Gb) to 69.8 million (8.72 Gb), with a mean of 58.5 ± 4.7 million. The number of Illumina reads in each treatment group was well balanced, with 871.97 million reads (109.04 Gb) in the control group and 881.70 million reads (110.08 Gb) in the hypoxia group (**Table 1**).

**Table 1:** Sequencing statistics for individual paired end reads from the RNA-Seq library from *G. eckloni*.

Control group	Raw reads	Clean reads	Clean bases (Gb)	Hypoxia group	Raw reads	Clean reads	Clean bases (Gb)
Ct_BL1	61152636	58339060	7.30	Hy_BL1	73054904	69832780	8.72
Ct_BL2	66399392	63664174	7.96	Hy_BL2	58689648	56160434	7.02
Ct_BL3	63096964	59724208	7.46	Hy_BL3	64921608	62405518	7.80
Ct_HT1	63675288	61983196	7.74	Hy_HT1	64270992	58629722	7.32
Ct_HT2	58567260	56934910	7.12	Hy_HT2	59945162	55668960	6.96
Ct_HT3	64550802	63070172	7.88	Hy_HT3	58874390	54440242	6.80
Ct_B1	60245488	58484922	7.32	Hy_B1	64107014	59203940	7.40
Ct_B2	57955130	56780800	7.10	Hy_B2	55709096	50899436	6.36
Ct_B3	54788786	53537580	6.70	Hy_B3	58714566	53933376	6.74
Ct_H1	55698856	54104572	6.76	Hy_H1	73159030	69184552	8.64
Ct_H2	64274636	62805786	7.86	Hy_H2	68307826	64420564	8.06
Ct_H3	55967440	54870948	6.86	Hy_H3	68961892	64287232	8.04
Ct_M1	58775520	57210602	7.16	Hy_M1	55676282	53147432	6.64
Ct_M2	54759762	53850208	6.74	Hy_M2	58281188	55359538	6.92
Ct_M3	57943000	56613202	7.08	Hy_M3	57161448	54126230	6.76
**Total**	897850960	871974340	109.04	**Total**	939835046	881699956	110.08

*Ct and Hy represent normoxia and hypoxia treatments, respectively. BL, blood; HT, Liver; B, brain; H, heart; M, muscle.*

As the genome of *G. eckloni* is not available, we assembled the transcripts *de novo*. *De novo* assembly analysis across all Illumina reads identified 551,430 transcript fragments, ranging in length from 201 to 34,873 bp, with an average length of 865 bp. The N50 and N90 values of the obtained transcripts were 1486 and 329 bp, respectively. Further assembly analysis identified 337,481 gene fragments across all transcripts, with an average length of 656 bp. The N50 and N90 values of the obtained gene fragments were 972 and 266 bp, respectively.

### Functional Annotation

To annotate the *G. eckloni* transcriptome, all 337,481 gene fragments were annotated against six public databases (Nr protein, Nt, Swiss-Prot, KEGG, GO, and COG) using BLASTX and BLASTN, both with an E-value cut-off of <10^-5^. We annotated 46,418 gene fragments against the Nr protein database (13.75%; **Table 2**). The E-value distribution of the top hits in the Nr database ranged from 0 to 1.0E^-15^ (**Supplementary Figure 1A**). The similarity distribution of the top BLAST hits for each sequence ranged from 18 to 100% (**Supplementary Figure 1B**). The most common species with similar sequences in the Nr database, were *Danio rerio* (62.1%), followed by *Astyanax mexicanus* (4.7%), *Oncorhynchus mykiss* (3.9%), *Oreochromis niloticus* (1.8%), and *Oryzias latipes* (1.5%) (**Supplementary Figure 1C**). The previously published genome annotations for these species are comprehensive and largely accepted, suggesting that the *G. eckloni* gene fragments were correctly assembled and annotated.

**Table 2:** Annotation of assembled *G. eckloni* gene fragments.

Category	Count	Percentage (%)^c^
Nr^a^ annotated sequences	46,418	13.75%
Nt^b^ database	117,993	34.96%
Swiss-Prot	34,524	10.22%
GO classified sequences	50,492	14.96%
COG classified sequences	21,106	6.25%
KEGG classified sequences	20,660	6.12%

*^*a*^Nr, NCBI non-redundant sequence database. ^*b*^Nt, NCBI nucleotide sequence database. ^*c*^Percentage of annotated sequences in total 337,481 assembled gene fragments of *G. eckloni*.*

**FIGURE 1:**
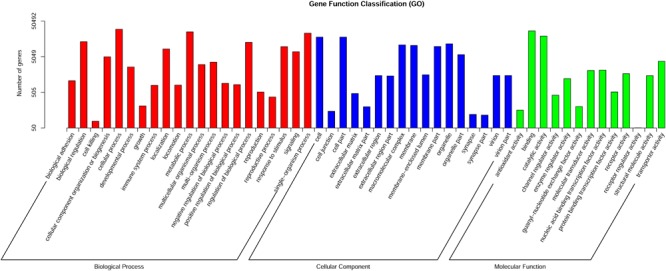
Gene ontology classification of putative gene functions in *Gymnocypris eckloni*. The transcripts are categorized into three main categories: biological process, cellular component, and molecular function.

Gene Ontology (GO) assignments were used to classify the functions of the predicted *G. eckloni* gene fragments. Based on sequence homology, 50,492 sequences were categorized into 51 functional groups in three functional divisions (**Figure 1**). Many of the gene fragments were classified as cellular processes, metabolic processes, cell, cell part, single-organism processes, binding, and catalytic activity.

**FIGURE 2:**
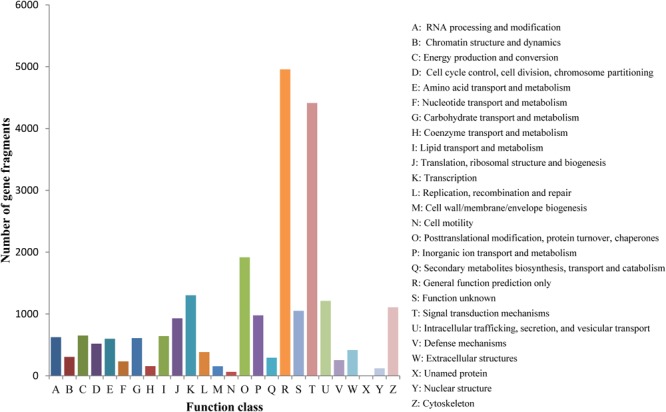
KOG classification of putative *Gymnocypris eckloni* proteins. The y-axis indicates the number of gene fragments in each functional cluster.

To test the effectiveness of the annotation process and evaluate the integrity of the transcriptome libraries, gene fragments were classified based on KOG categories. Of the 46,418 Nr hits, 21,106 sequences were assigned to 26 KOG categories (**Figure 2**). Of these, “General function prediction only” was assigned the most sequences (4,956), followed by “Signal transduction mechanisms” (4,412 sequences), “Posttranslational modification, protein turnover, chaperones” (1,917 sequences), “Transcription” (1,303 sequences), “Intracellular trafficking, secretion, and vesicular transport” (1,211 sequences), and “Translation, ribosomal structure and biogenesis” (929 sequences). The KOG categories “Cell motility” and “Unnamed protein” had the fewest sequences (64 and 3 sequences, respectively).

**FIGURE 3:**
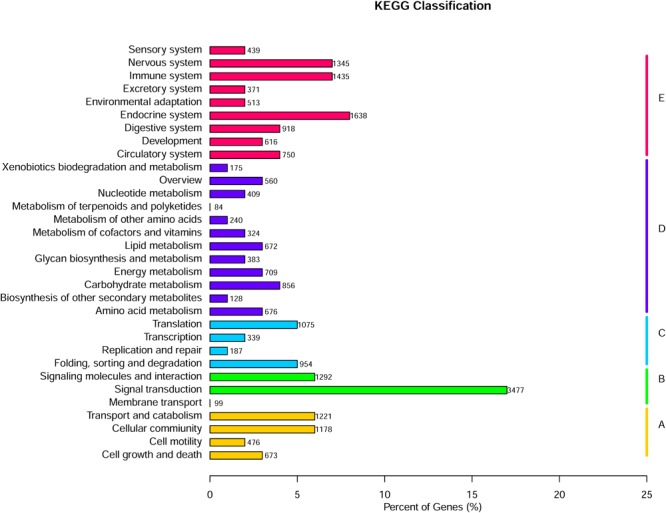
KEGG classification of *Gymnocypris eckloni* genes. A, cellular processes; B, environmental information processing; C, genetic information processing; D, metabolism; E, organismal systems.

The 46,418 annotated sequences were mapped to the canonical reference pathways in the KEGG database. Of these, 26,660 gene fragments were successfully assigned to 278 KEGG pathways in five categories (**Figure 3**). Most gene fragments fell into the KEGG pathway category organismal systems (8,025 sequences), followed by metabolic pathways (5,216 sequences), environmental information processing (4,868 sequences), cellular processes (3,548 sequences), and genetic information processing (2,555 sequences).

### DEGs

Differential expression analysis of gene fragments indicated that 462 sequences were differentially expressed in fish exposed to hypoxia. Of these, 28 were differentially expressed in liver (20 upregulated and 8 downregulated), 30 were differentially expressed in the heart (18 upregulated and 12 downregulated), 74 were differentially expressed in the blood (59 upregulated and 15 downregulated), 60 were differentially expressed in the brain (48 upregulated and 12 downregulated), and 282 were differentially expressed in the muscle (247 upregulated and 35 downregulated). To validate our Illumina sequencing results, 21 DEGs from liver, heart, brain, and muscle were selected for qRT-PCR analysis. The expression patterns of 20 of these genes were consistent with those indicated by our RNA-Seq analysis. The remaining DEG, polyprotein (Gene ID: c227508_g1), was upregulated according to the RNA-Seq analysis, but downregulated according to the qRT-PCR analysis (**Supplementary Figure 2**).

**FIGURE 4:**
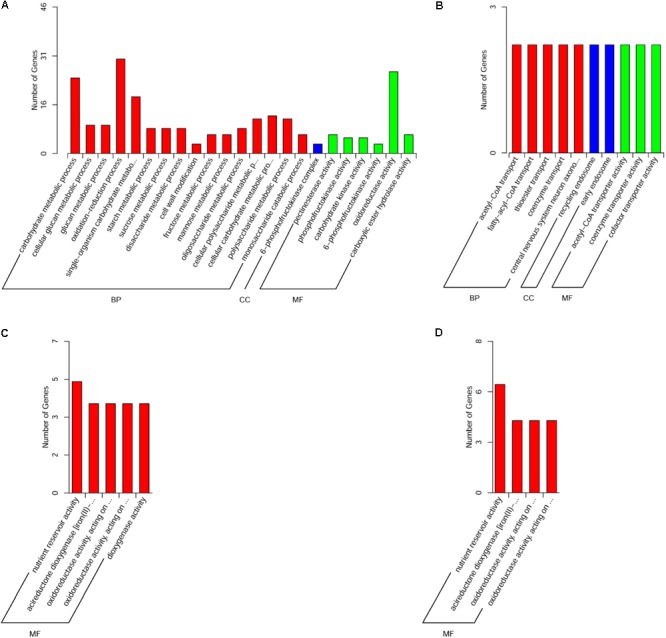
Gene ontology functional classification of **(A)** significantly upregulated genes in the muscle, **(B)** significantly downregulated genes in the muscle, **(C)** significantly upregulated genes in the liver, and **(D)** significantly upregulated genes in the brain. BP, biological process; CC, cellular component; MF, molecular function.

GO functional enrichment analysis using GO slim terms showed that only the DEGs in the muscle, liver, and brain were significantly enriched in GO terms (**Supplementary Table 2**). In the muscle, 231 of the 247 upregulated DEGs were significantly enriched in 22 biological process categories, one cellular component category, and six molecular function categories (**Figure 4A**), while 20 of the 35 downregulated DEGs were significantly enriched in five biological process categories, two cellular component categories, and three molecular function categories (**Figure 4B**). In the liver, the 20 upregulated DEGs were significantly enriched in five molecular function categories (**Figure 4C**). In the brain, 18 of the 48 upregulated DEGs were significantly enriched in four molecular function categories (**Figure 4D**).

**FIGURE 5:**
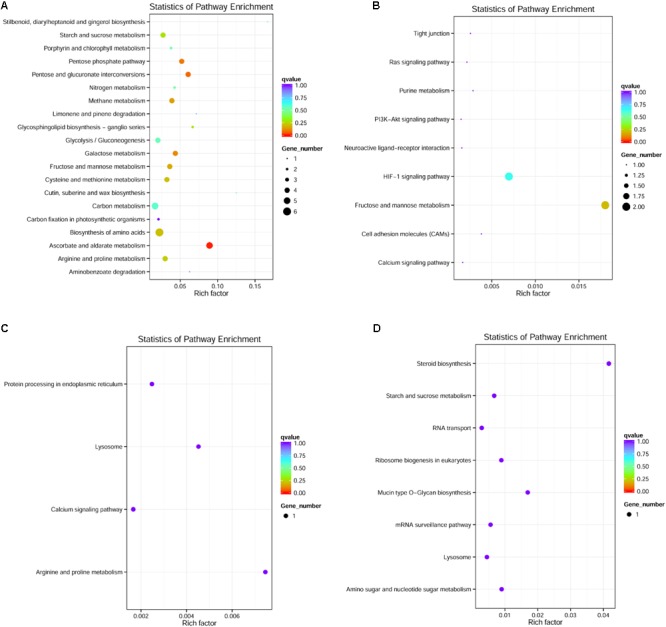
An overview of the KEGG pathways significantly enriched in differentially expressed genes in the **(A)** muscle, **(B)** blood, **(C)** brain, and **(D)** liver. The specific pathways are plotted along the y-axis, and the x-axis indicates the enrichment factor. The size of the colored dots indicates the number of significantly differentially expressed genes associated with each corresponding pathway: pathways with larger-sized dots contain a higher numbers of genes. The color of the each dot indicates the corrected *P*-value for the corresponding pathway.

Only DEGs in the muscle, liver, brain, and blood were enriched in KEGG pathways. For example, 60 significantly enriched KEGG pathways, including 119 DEGs, were identified in the muscle (see **Figure 5A** for the top 20 most enriched pathways; other enriched pathways are shown in **Supplementary Table 3**). The ascorbate and aldarate metabolism pathway, which is associated with antioxidant defense, was the most enriched pathway, followed by pentose and glucuronate interconversion, the pentose phosphate pathway, and galactose metabolism. In the blood, 11 DEGs were enriched in nine KEGG pathways (**Figure 5B**). In the brain, four DEGs were enriched in four KEGG pathways (**Figure 5C**). In the liver, seven DEGs were enriched in seven KEGG pathways (**Figure 5D**).

## Discussion

Limited oxygen availability (hypoxia) is an important environmental stressor that has a profound impact on the survival, reproduction, growth, and development of aquatic life, and thus the aquatic ecosystem as a whole (Shang and Wu, 2004; Storz et al., 2010). Many fish species are able to cope with or adapt to hypoxia via a complex suite of molecular strategies that operate at the transcriptional level (Gracey et al., 2001; van der Meer et al., 2005; Zhong et al., 2009; Kang et al., 2017; Liu et al., 2018). Here, we describe the transcriptomic response of five *G. eckloni* tissues to hypoxia, affording new insights into the adaptive mechanisms employed by fish subjected to hypoxic conditions.

Under hypoxia, fish may either reduce their metabolic rate to match reduced energy supply, or may maintain their metabolic rate, compensating for the ATP deficit with anaerobic metabolism (glycolysis) (Val et al., 1995; Gracey et al., 2001; Nikinmaa, 2002; Ton et al., 2003; Zhong et al., 2009). Here, several GO slim terms related to the aerobic metabolism of sugar, fat, and protein (including acetyl-CoA transport, fatty-acyl-CoA, coenzyme transport, acetyl-CoA transporter activity, and coenzyme transporter activity) were downregulated in the muscle of the hypoxic fish, as compared to the muscle of the normoxic fish. In addition, several GO slim terms related to the glycolysis pathway were upregulated in the muscle of the hypoxic fish as compared to the muscle of the normoxic fish, including the 6-phosphofructokinase complex, phosphofructokinase activity, and 6-phosphofructokinase activity. These enriched GO terms were supported by our KEGG pathway analysis. These results were consistent with the expression patterns of these genes in several other fish species (Gracey et al., 2001; Nikinmaa and Rees, 2005; Zhong et al., 2009).

The downregulation of genes associated with the tricarboxylic acid cycle (TCA) concurrent with the upregulation of genes associated with the glycolysis pathway in the muscle of hypoxic fish indicated a shift from aerobic oxidation to anaerobic glycolysis. However, glycolysis pathway genes were not upregulated in other tissues, possibly because muscle tissue has a higher glycolytic capacity than other tissues, and is thus better suited to supply the reduced metabolic needs of hypoxic *G. eckloni*. In addition, three genes involved in the pentose phosphate pathway were induced in the muscle of hypoxic fish, suggesting that muscle tissue may use pyrophosphate (PPi) as an energy source for hypoxic metabolism in *G. eckloni*.

Catabolism of gluconeogenic amino acids produces either pyruvate or an intermediate of the TCA cycle to generate carbon skeletons for gluconeogenesis (Gracey et al., 2001). Here, two amino acid pathways (“cysteine and methionine metabolism” and “arginine and proline metabolism”) were enriched in the muscle. Several genes involved these two pathways were upregulated under hypoxia, including S-adenosylmethionine synthase, S-adenosylmethionine decarboxylase, brachypodium distachyon adenosylhomocysteinase, homocysteine methyltransferase, and prolyl 4-hydroxylase.

Glucose-6-phosphatase (G-6-Pase) was strongly expressed in the liver, and it was accompanied by hypoxia-induced gluconeogenesis. G-6-Pase catalyzes the dephosphorylation of glucose-6-phosphate to glucose, which is then circulated to other tissues to fuel glycolysis (Gracey et al., 2001; Zhong et al., 2009).

It is notable that the expression of 6-phosphofructo-2-kinase/fructose-2, 6-biphosphatase (PFK-2) was downregulated in the blood under hypoxia, which is not consistent with observations in the muscles and livers of other fish (Gracey et al., 2001; Zhong et al., 2009). PFK-2 may contribute to the regulation of glycolytic flux under hypoxia (Yalcin et al., 2009; Zhong et al., 2009). Thus, in *G. eckloni*, amino acid catabolism coupled with gluconeogenesis in the liver, concurrent with a decrease in the transcriptional activity of PFK-2, may represent an alternative mechanism for the maintenance of blood glucose levels during hypoxia.

In the muscle, several genes involved in amino acid catabolism (e.g., S-adenosylmethionine synthase and S-adenosylmethionine decarboxylase) were upregulated under hypoxia. S-adenosylmethionine synthase and S-adenosylmethionine decarboxylase catalyze stages of methionine degradation. Consistent with the upregulation of these two genes, glutathione metabolism, which plays an important role in ammonia detoxification in the muscle (Gracey et al., 2001), increased significantly under hypoxia.

Studies have shown that toxic reactive oxygen species (ROS) are overproduced in fish muscle under hypoxic conditions (Lushchak et al., 2001; Bagnyukova et al., 2007; Clanton, 2007). Indeed, the activity levels of antioxidant enzymes, such as catalase, glutathione peroxidase, and superoxide dismutase, increase significantly to protect fish exposed to hypoxic stress (Lushchak et al., 2001; Zhong et al., 2009; Huang et al., 2015; Zhang et al., 2016; Wang et al., 2017). It is interesting that the key pathway related to the antioxidant defense system, ascorbate and aldarate metabolism, was significantly enriched in the muscle of *G. eckloni*, and that muscle was most affected by hypoxic stress. Three genes involved this pathway were upregulated under hypoxia: L-ascorbate oxidase, L-ascorbate peroxidase, and GDP-L-galactose phosphorylase. Both L-ascorbate oxidase and L-ascorbate peroxidase are oxidoreductases. The former acts on diphenols and related substances as the donor, with oxygen as the acceptor, while the latter acts on peroxides as the acceptor (peroxidases). They are the most important enzymes in ascorbic acid-glutathione detoxification, playing an important role in scavenging ROS and protecting cells from the destructive effects of ROS.

Consistent with this increase in ascorbate and aldarate metabolism, GDP-L-galactose phosphorylase, the key enzyme for ascorbic acid synthesis, was upregulated in the hypoxic *G. eckloni*. In addition, DyP-type peroxidase, a member of the haem peroxidase family, was upregulated in muscle, indicating the detoxification of the muscle exposed to hypoxic stress. DyP-type peroxidase degrades typical peroxidase substrates, as well as hydroxyl-free anthraquinone. Hypoxic conditions induce the expression of genes involved in the cellular stress response, such as heat shock proteins (HSPs). Ischemia-induced HSP70 expression has been demonstrated in the tissues of intact animals (Dillmann et al., 1986), and in cultured mammalian cells (Benjamin et al., 1990). HSP70 functions as a chaperone, protects cells from apoptosis, and plays important roles in reducing intracellular oxygen free radicals and maintaining the intracellular redox state (Fang et al., 2012). During hypoxia, regulation of the antioxidant system may be a biochemical mechanism that minimizes the destructive effects of free radicals (Zhong et al., 2009). While the enzymes involved in the antioxidant system differ somewhat among fish species, the activation of these enzymes also varies dependent on hypoxia regime.

There was significantly more insulin-like growth factor binding protein 1 (IGFBP-1) mRNA in the blood of *G. eckloni* under hypoxia as compared to the blood under normoxia. IGFBP-1 is a negative regulator of insulin and insulin-like growth factor in circulating blood (Gracey et al., 2001). Studies have shown that IGFBP-1 upregulation is correlated with intrauterine growth restriction in fetuses subjected to long-term chronic hypoxia (Chard, 1994); IGFBP-1 is also involved in the suppression of cell growth and proliferation in the euryoxic fish *Gillichthys mirabilis* after exposure to hypoxia (Gracey et al., 2001).

The ras-related and estrogen-regulated growth inhibitor-like protein (RERG), which negatively regulates the growth of cells involved in cardiac muscle development, was strongly upregulated in the heart of *G. eckloni* under hypoxia. In contrast, eukaryotic elongation factor 2 kinase (eEF-2K) was downregulated in *G. eckloni* muscle under hypoxia. eEF-2K is a dedicated kinase that regulates the activity of eukaryote extension factor 2 (eEF-2), its only known substrate, via phosphorylation at Thr-56 (Ryazanov et al., 1988; Dumont-Miscopein et al., 1994; Tavares et al., 2014). This decreases the affinity of phospho-eEF-2 for the ribosome, which impedes the elongation phase of protein translation, inhibiting the activity of eEF-2 (Carlberg et al., 1990; Dumont-Miscopein et al., 1994).

Interestingly, translationally-controlled tumor protein (TCTP) was also upregulated in the muscle. TCTP has similar properties to tubulin binding proteins, and associates with microtubules in a cell cycle-dependent manner (Bommer and Thiele, 2004). The overexpression of TCTP results in cell growth retardation (Gachet et al., 1999). Therefore, under hypoxia, the expression patterns of genes involved in the suppression of cell growth and proliferation changed, and protein synthesis rates decreased. These mechanisms may represent an especially important energy-saving strategy for the survival of *G. eckloni* in hypoxic environments.

Hypoxia-inducible factors (HIFs) have been recognized as the master regulators of the hypoxia signaling pathway; HIFs play important roles in maintaining dynamic oxygen balance and regulating changes in oxygen concentration under hypoxia (Xiao, 2015). An increase in Hif-1α/2α mRNA expression in response to hypoxia has been observed in mouse skeletal muscle (Stroka et al., 2001), in various tissues of *Bos grunniens* (Wang et al., 2006), and the liver and gill of *Megalobrama amblycephala* (Li et al., 2015). However, we detected no changes in the expression of Hif-1α/2α in *G. eckloni* subjected to hypoxia. Instead, egl nine homolog 1 (EGLN1) was significantly upregulated in the brain of *G. eckloni*. EGLN1 is considered homologous to prolyl-hydroxylase domain-containing protein 2 (PHD2), which plays an important role in the stabilization of Hif-1α. It is thus clear that the response of the HIF signaling pathway to hypoxia differs between mammals and fish. The HIF response also appears to vary among teleosts depending on species, hypoxia regime, and tissue. Further investigation is required to fully understand these variations.

## Conclusion

We sequenced, assembled, and characterized the transcriptome of the Tibetan schizothoracine fish *G. eckloni*. Hypoxia had a greater effect on muscle than on any other tissue. Under hypoxia, *G. eckloni* underwent changes in gene expression, suggesting several key adaptive strategies. First, a reorganization of metabolic pathways and a transition from aerobic oxidation to anaerobic glycolysis. Second, a suppression of major energy-requiring processes, such as cell growth/proliferation and protein synthesis. In addition to these two strategies, which have also been observed in other teleosts, *G. eckloni* strengthens its antioxidant system and minimizes ischemic injury in response to hypoxia. This study is the first to analyze gene expression patterns in the Tibetan schizothoracine fish *G. eckloni* in response to hypoxic stress. Nevertheless, further studies using additional physiological and molecular approaches are necessary to verify the transcriptomic response of schizothoracine fish to hypoxia.

## Author Contributions

DQ designed the study and wrote the manuscript. YC and ZZ conducted the hypoxia treatment and collected the materials. RW, MX, and QC performed the experiments and analyzed the data. All authors have read and approved the final manuscript.

## Conflict of Interest Statement

The authors declare that the research was conducted in the absence of any commercial or financial relationships that could be construed as a potential conflict of interest.

## Footnotes

1 http://www.genome.jp/kegg/kaas/
