# Supplementary material for: Transcriptome Analysis Provides Insights Into the Adaptive Responses to Hypoxia of a Schizothoracine Fish (Gymnocypris eckloni)
Source: Front Physiol. 2018 Sep 21;9:1326. doi: 10.3389/fphys.2018.01326 (PMC6160557; doi:10.3389/fphys.2018.01326)
Supplement: Supplementary file 1 [file Table_1.DOC]

Supplementary Table 1 Primers used for the qRT-PCR analysis of differently expressed genes in the *G. eckloni*.

| Gene ID | Description | Primer | Tissue |
| --- | --- | --- | --- |
| c220274_g1 | solute carrier family 26 member 10 | GGGTTCAGTGGTGGAGCAA  CGCCTTGACAATCGGTTCT | Brain |
| c103332_g1 | putative germin-like protein 2-1-like | GCTTCTTCCCTTTGTCTTTGTT  CGAATAAGTTACTGGTGTTGCC | Brain |
| c236251_g1 | Retrovirus-related Pol polyprotein from type-1 retrotransposable element R2 | TACATCTGGGAGTCTGCTGCT  ACGAAGAGCGTAATGGGGTAG | Brain |
| c220646_g1 | ATPase, Ca++ transporting, cardiac muscle, fast twitch 1 like isoform X1 | CCTCCACGCTCCGCCAAAGA  CAGCCACAGTAGCAGCACCCACAT | Brain |
| c246699_g1 | Probable RNA-directed DNA polymerase from transposon | AAGATACCTTGTGCGTCGTGA  CCATACCATTCTACAGCCAGC | Brain |
| c238624_g1 | secretogranin II precursor | GCTTGAGAAGCTGTTTTGGTG  GGCTGTGAGAAGTTGCGTTTA | Brain/Liver |
| c195360_g1 | chondroadherin-like protein | TTCAGGTGGTGTCCCTTCAT  GAGTTTAGTCGCTGCTGTGCT | Liver |
| c237365_g1 | lathosterol oxidase | TCTGCCCTTTGATTTGATGTC  TTGCTGTGGTTGGAGTTTTTC | Liver |
| c238791_g1 | Reverse transcriptase | GGAAAACGATGTAGACGAGCA  ATCTGCCTAACCCCTATCT GG | Liver |
| c202838_g2 | chymotrypsin B1 precursor | TCAGCGGGTATCAAAGTCATC  ATCATCCATCTGCTCC TGTGT | Liver |
| c246838_g1 | Voltage-gated potassium channel subunit beta-2 | TCGTTATGGGAGTCCAAAGG  CCGAGAAGCATTTCAGCCT | Heart |
| c228321_g1 | fos-related antigen 2 isoform X2 | CCATCCCTGCCTCCAACTA  AGACTAACCTGCTCATCTCGCT | Heart |
| c227508_g1 | Polyprotein | AGTGGGAGACTTGAGATGCCT  ATGTTTTGATACGTGGTTGGG | Heart |
| c239008_g1 | NXPE family member 3-like isoform X3 | TCAATCAGGGCCACTCATAGCA  TGGGTGAAGAAATCCAGAGGGTA | Heart |
| c175553_g1 | Integrase core domain//RNase H | CATTGTCAGGGAAGATAATAGGAAC  TTGCTTCATTGATGGAGGGA | Heart |
| c191596_g1 | Protein TAR1 | TAGTAACGGCGAGCGAACC  AGGACGCTTCTCCAGACTACAA | Muscle |
| c233064_g1 | eukaryotic elongation factor 2 kinase | GCTGCCACATCACATTCTACC  GGCGTGTTTCCAGTCCATAA | Muscle |
| c94099_g2 | pectate lyase | GCCTTGGCGTCAAAATCG  ATCGGGTTGGTGGCATAGC | Muscle |
| c224746_g1 | bcl-2-like protein 15-like | ACTGGGTGACGATTATGATGAGA  AGACCACTTCCGCTCTTTCC | Muscle |
| c235751_g1 | dnaJ homolog subfamily A member 1 | GCTCGGCGTCAGTCCTAAA  GCTTCCACCACCAAAGAACAT | Muscle |

Supplementary Table 2 The GO terms of significantly expressed genes in *G. eckloni*.

| **GO slim term of significantly upregulated genes in muscle** | **GO_ID** | **Gene number** |
| --- | --- | --- |
| **Biological process** |  |  |
| Carbohydrate metabolic process | GO：0005975 | 24 |
| Cellular glucan process | GO：0006073 | 9 |
| Glucan metabolic process | GO：0044042 | 9 |
| Oxidation-reduction process | GO：0055114 | 30 |
| Single-organism carbohydrate metabolic process | GO：0044723 | 18 |
| Starch metabolic process | GO：0005982 | 8 |
| Sucrose metabolic process | GO：0005985 | 8 |
| Disaccharide metabolic | GO：0005984 | 8 |
| Cell wall modification | GO：0042545 | 3 |
| Fructose metabolic process | GO：0006000 | 6 |
| Mannose metabolic process | GO：0006013 | 6 |
| Oligosaccharide metabolic | GO：0009311 | 8 |
| Cellular polysaccharide metabolic process | GO：0044264 | 11 |
| Cellular carbohydrate metabolic process | GO：0044262 | 12 |
| Polysaccharide metabolic process | GO：0005976 | 11 |
| Monosaccharide catabolic process | GO：0046365 | 6 |
| **Cellular component** |  |  |
| 6-phosphofructokinase complex | GO：0005945 | 3 |
| **Molecular function** |  |  |
| Pectinesterase activity | GO：0030599 | 6 |
| Phosphofructokinase activity | GO：0008443 | 5 |
| Carbohydrate kinase activity | GO：0019200 | 5 |
| 6-phosphofructokinase activity | GO：0003872 | 3 |
| Oxidoreductase activity | GO：0016491 | 26 |
| Carboxylic ester hydrolase activity | GO：0052689 | 6 |
| **GO slim term of significantly downregulated genes in muscle** | **GO_ID** | **Gene number** |
| **Biological process** |  |  |
| Acetyl-coa transport | GO：0015876 | 2 |
| Fatty-acyl-coa | GO：0015916 | 2 |
| Thioester transport | GO：1901337 | 2 |
| Coenzyme transport | GO：0051182 | 2 |
| Central nervous system neuron axonogenesis | GO：0021955 | 2 |
| **Cellular component** |  |  |
| Recycling endosome | GO：0055037 | 2 |
| Early endosome | GO：0005769 | 2 |
| **Molecular function** |  |  |
| Acetyl-coa transporter activity | GO：0008521 | 2 |
| Coenzyme transporter activity | GO：0051185 | 2 |
| Cofactor transporter activity | 辅GO：0051184 | 22 |
| **GO slim term of significantly upregulated genes in liver** | **GO_ID** | **Gene number** |
| **Molecular function** |  |  |
| Nutrient reservoir activity | GO：0045735 | 4 |
| Acireductone dioxygenase [iron(II)-requiring] activity | GO：0010309 | 4 |
| Oxidoreductase activity, acting on single donors with incorporation of molecular oxygen, incorporation of two atoms of oxygen | GO：0016702 | 4 |
| Oxidoreductase activity, acting on single donors with incorporation of molecular oxygen | GO：0016701 | 4 |
| Dioxygenase activity | GO：0051213 | 4 |
| **GO slim term of significantly upregulated genes in brain** | **GO_ID** | **Gene number** |
| **Molecular function** |  |  |
| Nutrient reservoir activity | GO：0045735 | 6 |
| Acireductone dioxygenase [iron(II)-requiring] activity | GO：0010309 | 4 |
| Oxidoreductase activity, acting on single donors with incorporation of molecular oxygen, incorporation of two atoms of oxygen | GO：0016702 | 4 |
| Oxidoreductase activity, acting on single donors with incorporation of molecular oxygen | GO：0016701 | 4 |

Supplementary Table 3 Enriched KEGG pathways of differentially expressed genes in *G. eckloni*.

| **KEGG pathway** | **KO ID** | **Number of DEGs** |
| --- | --- | --- |
| **Significantly upregulated genes in blood** |  |  |
| Purine metabolism | ko00230 | 1 |
| Ras signaling pathway | ko04014 | 1 |
| Calcium signaling pathway | ko04020 | 1 |
| Neuroactive ligand-receptor interactio | ko04080 | 1 |
| PI3K-Akt signaling pathway | ko04151 | 1 |
| **Significantly downregulated genes in blood** |  |  |
| Fructose and mannose metabolism | ko00051 | 2 |
| HIF-1 signaling pathway | ko04066 | 2 |
| Cell adhesion molecules (CAMs) | ko04514 | 1 |
| Tight junction | ko04530 | 1 |
| **Significantly upregulated genes in brain** |  |  |
| Lysosome | ko04142 | 1 |
| Protein processing in endoplasmic reticulum | ko04141 | 1 |
| **Significantly downregulated genes in blood** |  |  |
| Arginine and proline metabolism | ko00330 | 1 |
| Calcium signaling pathway | ko04020 | 1 |
| Metabolic pathways | ko01100 | 1 |
| **Significantly upregulated genes in muscle** |  |  |
| Ascorbate and aldarate metabolism | ko00053 | 5 |
| Pentose and glucuronate interconversions | ko00040 | 4 |
| Pentose phosphate pathway | ko00030 | 4 |
| Galactose metabolism | ko00052 | 4 |
| Methane metabolism | ko00680 | 4 |
| Fructose and mannose metabolism | ko00051 | 4 |
| Biosynthesis of amino acids | ko01230 | 6 |
| Cysteine and methionine metabolism | ko00270 | 4 |
| Arginine and proline metabolism | ko00330 | 4 |
| Starch and sucrose metabolism | ko00500 | 4 |
| Nitrogen metabolism | ko00910 | 2 |
| Glycolysis / Gluconeogenesis | ko00010 | 4 |
| Stilbenoid, diarylheptanoid and gingerol biosynthesis | ko00945 | 1 |
| Carbon metabolism | ko01200 | 5 |
| Cutin, suberine and wax biosynthesis | ko00073 | 1 |
| Limonene and pinene degradation | ko00903 | 1 |
| Aminobenzoate degradation | ko00627 | 1 |
| Carbon fixation in photosynthetic organisms | ko00710 | 2 |
| Photosynthesis - antenna proteins | ko00196 | 1 |
| Oxidative phosphorylation | ko00190 | 4 |
| Selenocompound metabolism | k00549 | 1 |
| Base excision repair | k10802 | 1 |
| Glycosaminoglycan biosynthesis | k01793 | 1 |
| HIF-1 signaling pathway | K09592 K04372 | 2 |
| Porphyrin and chlorophyll metabolism | K00228 | 1 |
| Biosynthesis of unsaturated fatty acids | K10256 | 1 |
| Glycerophospholipid metabolism | K01049 K15728 | 2 |
| Glyoxylate and dicarboxylate metabolism | K01602 | 1 |
| Adherens junction | K04490 K05700 | 2 |
| Glutathione metabolism | K00434 | 1 |
| Amino sugar and nucleotide sugar metabolism | K00963 | 1 |
| Glycerolipid metabolism | K15728 | 1 |
| [Fatty acid metabolism](../../../../C:%5C科研文件夹%5C文件夹3%5Cdata_give_20150106%5CNHT140199_Gymnocypris_Przewalskii_result%5CNHT140199_Gymnocypris_Przewalskii_results%5C12.DEG_KEGGenrichment%5CMuscle%5CHy_MvsCt_M_up_kegg_web%5Csrc%5Cko01212.html) | K10256 | 1 |
| **Significantly downregulated genes in muscle** |  |  |
| Glycosphingolipid biosynthesis - ganglio series | ko00604 | 2 |
| Porphyrin and chlorophyll metabolism | ko00860 | 1 |
| Glutathione metabolism | ko00480 | 1 |
| Proteasome | ko03050 | 1 |
| Glycerolipid metabolism | ko00561 | 1 |
| Adherens junction | ko04520 | 1 |
| Tight junction | ko04530 | 1 |
| Ras signaling pathway | ko04014 | 1 |
| PI3K-Akt signaling pathway | ko04151 | 1 |
| **Significantly upregulated genes in liver** |  |  |
| Steroid biosynthesis | ko00100 | 1 |
| Biosynthesis of secondary metabolites | ko01110 | 2 |
| Amino sugar and nucleotide sugar metabolism | ko00520 | 1 |
| Ribosome biogenesis in eukaryotes | ko03008 | 1 |
| Starch and sucrose metabolism | ko00500 | 1 |
| mRNA surveillance pathway | ko03015 | 1 |
| RNA transport | ko03013 | 1 |
| Metabolic pathways | ko01100 | 2 |
| **Significantly downregulated genes in liver** |  |  |
| Mucin type O-Glycan biosynthesis | ko00512 | 1 |
| Lysosome | ko04142 | 1 |
| Metabolic pathways | ko01100 | 1 |

A B

C

**Supplementary Figure 1** E value distribution in NR database (A), sequence similarity distribution (B) and unigene similarity analysis between *G. eckloni* and other fish species (C).


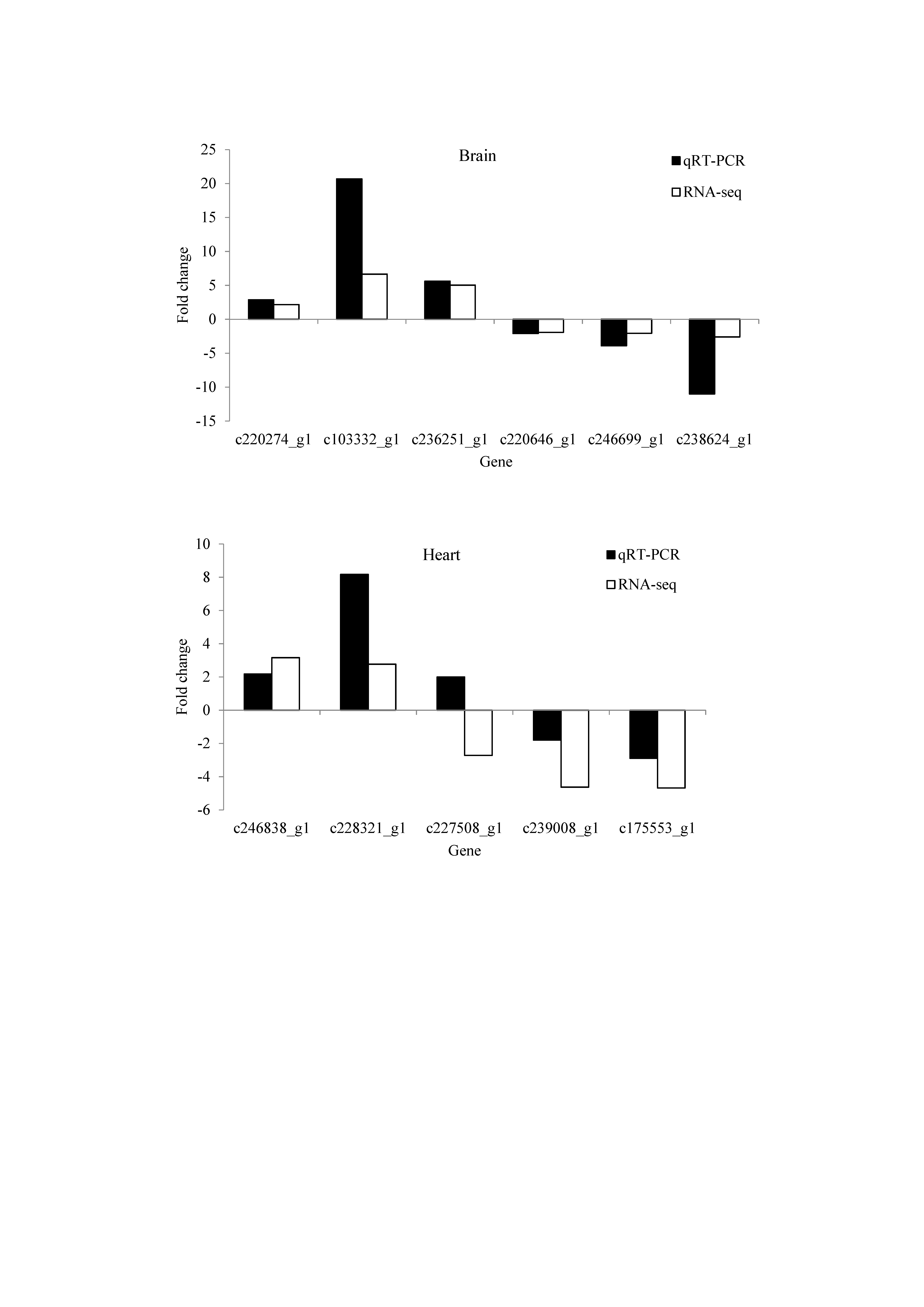

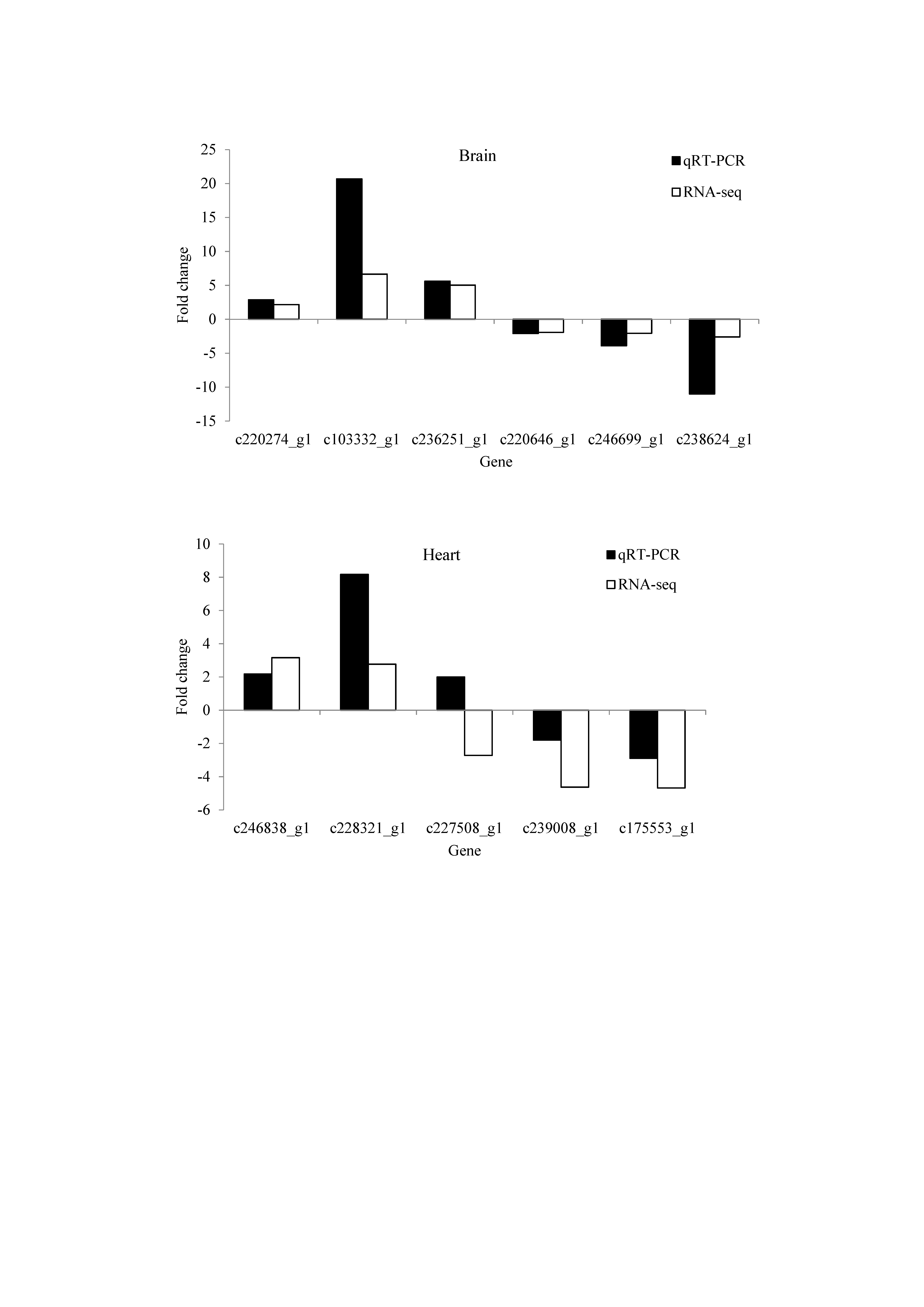


A B


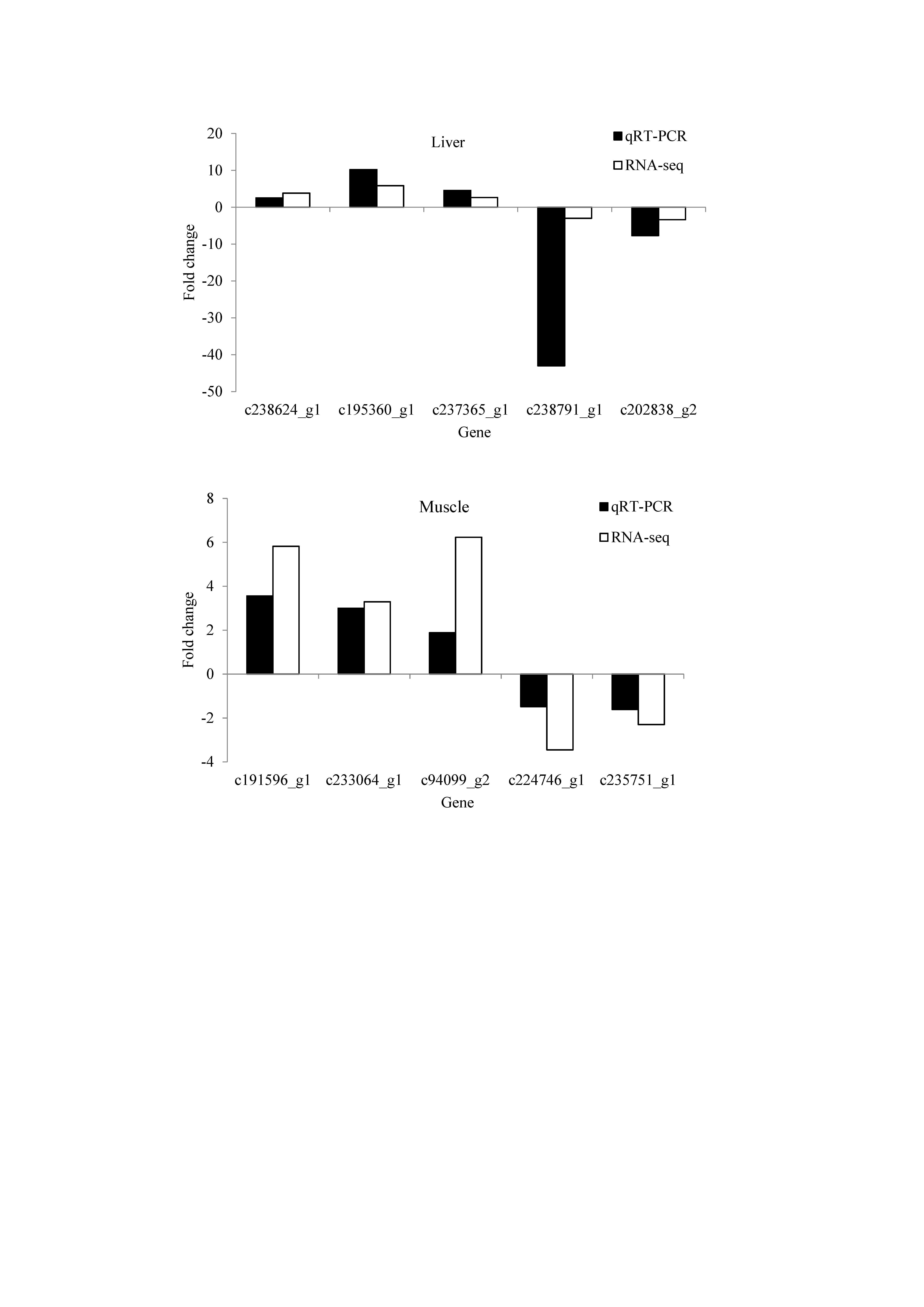

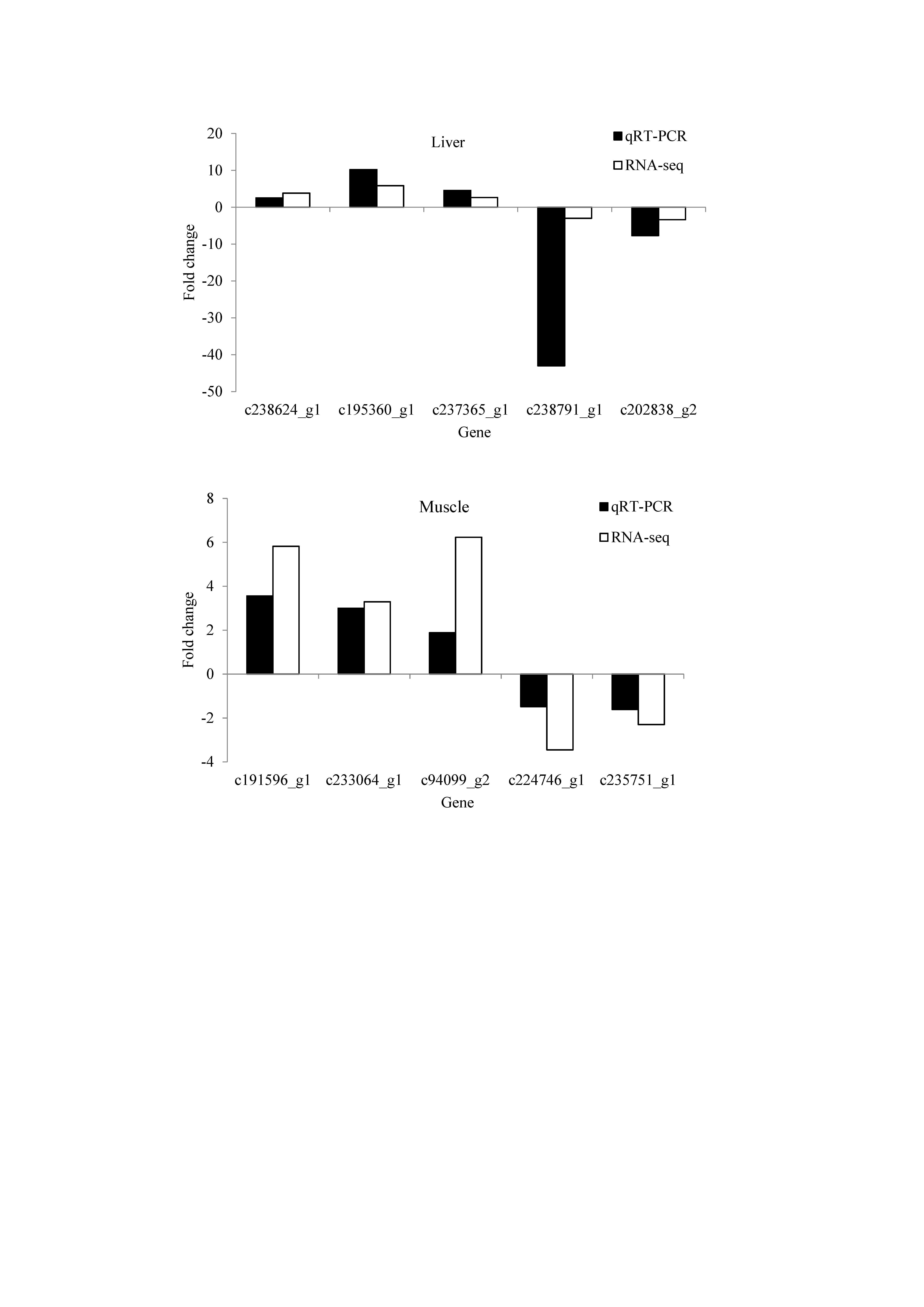


C D

**Supplementary Figure 2** qRT-PCR validation of the expression of selected genes in brain (A), heart (B), liver (C) and muscle (D).
